# Colony entropy—Allocation of goods in ant colonies

**DOI:** 10.1371/journal.pcbi.1006925

**Published:** 2019-08-05

**Authors:** Efrat Greenwald, Jean-Pierre Eckmann, Ofer Feinerman

**Affiliations:** 1 Department of Physics of Complex systems, Weizmann Institute of Science, Rehovot, Israel; 2 Département de Physique Théorique and Section de Mathématiques, University of Geneva, Geneva, Switzerland; Santa Fe Institute, UNITED STATES

## Abstract

Allocation of goods is a key feature in defining the connection between the individual and the collective scale in any society. Both the process by which goods are to be distributed, and the resulting allocation to the members of the society may affect the success of the population as a whole. One of the most striking natural examples of a highly successful cooperative society is the ant colony which often acts as a single superorganism. In particular, each individual within the ant colony has a “communal stomach” which is used to store and share food with the other colony members by mouth to mouth feeding. Sharing food between communal stomachs allows the colony as a whole to get its food requirements and, more so, allows each individual within the colony to reach its nutritional intake target. The vast majority of colony members do not forage independently but obtain their food through secondary interactions in which food is exchanged between individuals. The global effect of this exchange is not well understood. To gain better understanding into this process we used fluorescence imaging to measure how food from a single external source is distributed and mixed within a *Camponotus sanctus* ant colony. Using entropic measures to quantify food-blending, we show that while collected food flows into all parts of the colony it mixes only partly. We show that mixing is controlled by the ants’ interaction rule which implies that only a fraction of the maximal potential is actually transferred. This rule leads to a robust blending process: *i.e*., neither the exact food volume that is transferred, nor the interaction schedule are essential to generate the global outcome. Finally, we show how the ants’ interaction rules may optimize a trade-off between fast dissemination and efficient mixing. Our results regarding the distribution of a single food source provide a baseline for future studies on distributed regulation of multiple food sources in social insect colonies.

## Author summary

We study how food is distributed in colonies of ants. Food collected by a small fraction of ants is distributed throughout the colony through a series of mouth-to-mouth interactions. An interesting interplay exists between food dissemination and food mixing within the colony. High levels of dissemination are important as they ensure that any food type is available to any ant. On the other hand, high dissemination induces homogenization which reduces the required variety of nutritional choices within the colony. Tracking fluorescent-labelled food and interpreting the results within the concepts of information theory, we show that food collected by each forager reaches almost every ant in the colony. Nonetheless, it is not homogenized across workers, resulting in a limited level of mixing. We suggest that the difference in food mixture held by each individuals can provide ants with the potential to control their nutritional intake by interacting with different partners.

## Introduction

Food sharing in social insects is a compelling example of cooperation within a population [1–7]. Ants and bees can store a considerable amount of liquids in a pre-digestion storage organ called the ‘crop’ [8–10]. The stored food can later be regurgitated and passed on to others by mouth-to-mouth feeding (oral trophallaxis) [10–12]. Trophallaxis is a principal mechanism of food-transfer between individuals and therefore, the crop is often referred to as a “social stomach” [8].

When food is exchanged through trophallaxis, it is stored within the crop of the recipient workers and mixed with the rest of food in the crop [13–17]. Food blending is therefore an important factor in any process mediated by trophallaxis: from nutrient transfer and the maintenance of gestalt odor to hormonal regulation and information sharing [8, 13, 18, 19]. The extent to which food is blended in the colony has only been partially addressed before [3, 14, 20–22] and is still an open question.

Food blending is especially interesting in light of the fact that most colony members do not leave the nest [5, 14, 16, 23, 24], and all food is brought in by a a small fraction of workers called the foragers [16, 25]. The inter-relations between food-supplies brought in by different foragers can be expected to have an important role in the nutritional regulation of the colony. Social insect colonies have a documented ability to tightly regulate both the global nutritional intake [15, 21] and the dissemination of food to various sub-populations (such as nurses, larvae and brood) which may have different nutritional needs [5, 14, 16, 23, 26, 27]. The mechanisms that underlie this regulation are, however, not fully understood [28].

**Fig 1:**
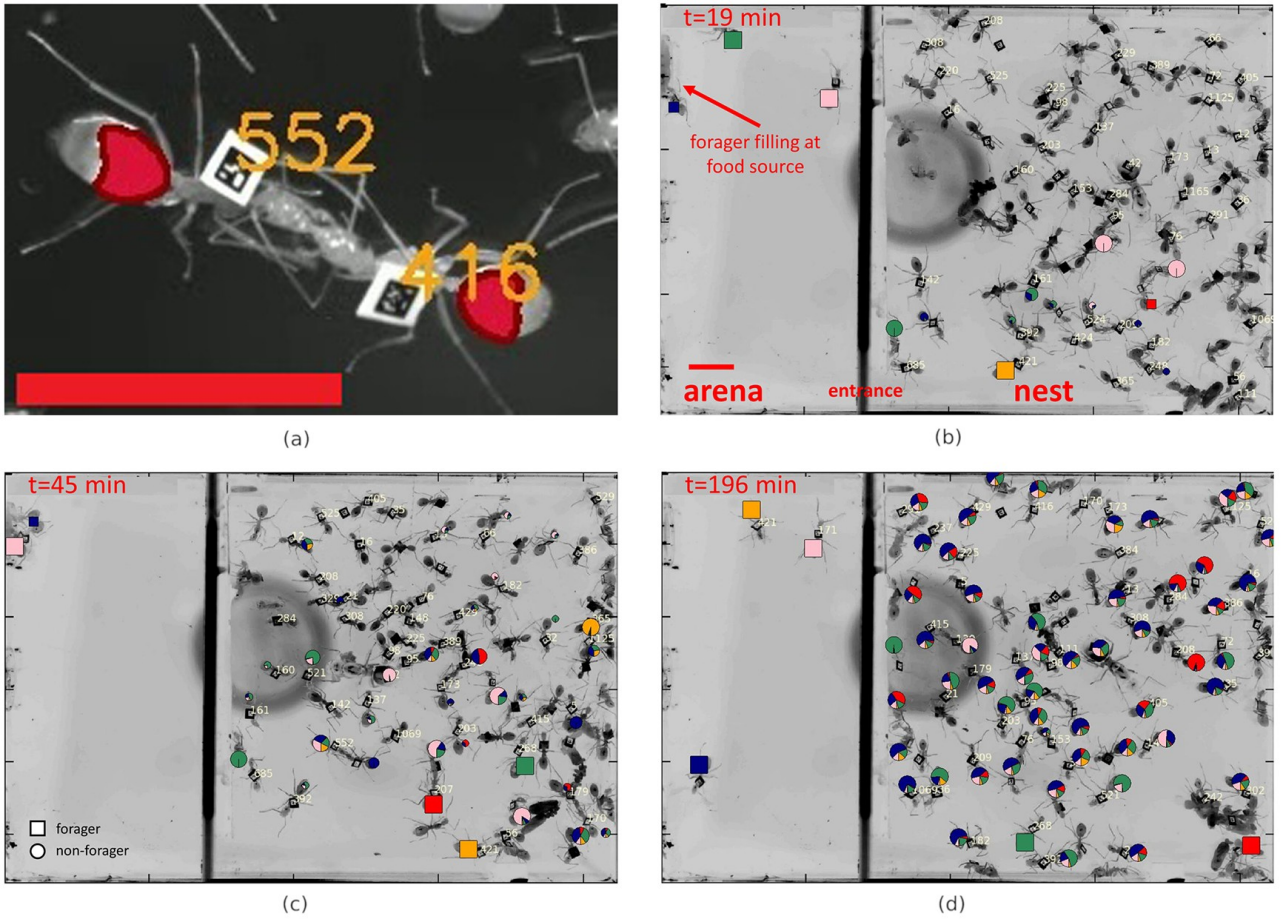
Quantifying food distribution within an ant colony by combining single ant tracking with fluorescent imaging. **a)** Two tagged workers engaged in trophallaxis. The identity of ants (orange numbers) was determined using Bugtag barcodes. The volume of food in the ants’ crop is measured using fluorescence imaging and overlaid in red. **b-d)** Food distribution across the colony and at different stages of the experiment. Markers (round: non-forager, square: forager) overlaid on ants depict their crop contents. Marker size is proportional to the food load held by each ant: *P*_*a*_ (small markers were set to a minimal size for clarity). Color division in markers of all ants depicts the computationally derived proportions of food in their crops according to the forager that first collected it (‘food-types’): (*P*(*f*|*A* = *a*)). Scale bars are 1cm. See also supplemental movie “Food dissemination in ant colony”.

Trophallactic food exchange requires physical contact between ants. The dissemination process is therefore conveniently described by a time ordered network, in which ants are the nodes and the food transfers are the (directed) edges. The topology of this network provides the underlying infrastructure of the food-sharing process [17, 29–31]. In the study of social insects and other real-world networks, the topology of the network can frequently be traced while the details of particular interactions are concealed [32, 33]. Indeed, previous studies that traced individuals in a colony have mainly focused either on the network topology [29, 31] or on coarse grained descriptions of food dissemination [1, 16, 22, 26, 27, 34]. In this study we use single ant identification and fluorescently-labeled food (Fig 1) to measure not only the interaction network but also the flow of food over this network. For technical reasons, these experiments are conducted with a single food source. Characterization of this basic case is a first but necessary step towards more complex scenarios which include multiple sources.

The flow of food is limited by capacity: As the crop of ants is of finite size, this imposes a constraint on the amount of food that can be transferred in an interaction. This physical constraint limits the rate of mixing as ants become more and more full. Therefore, a potential trade-off between fast rate of food accumulation and well mixed outcome is expected.

The main objective of our study is using single ant measurement techniques to quantify how food brought in by different foragers blends as it is being disseminated across an ant colony. To this end, we use Shannon entropy to quantify the quality of mixing in an ant’s crop. The Shannon entropy provides a single quantity that reflects the relative abundances of multiple constituents [35] and therefore sets a common scale by which food homogenization can be evaluated from our empirical data. Using our detailed measurements we characterize the interaction network and the rules by which food flows across this network. We then use hybrid simulations to identify which of these characteristics function as regulators of food mixing, and which might play a lesser role. Finally, we employ a theoretical model to study the trade-offs between food dissemination and nutritional homogenization.

## Results

### Food dissemination

We studied food (sucrose solution [80*g*/*l*]) dissemination in *Camponotus sanctus* ant colonies residing in an artificial, single chamber nest and following famine relief (see Materials and methods, Experimental Setup). The dissemination process begins when the foragers, a small subgroup of the ants which we label $F=\{1,2,\ldots ,N_{\text{foragers}}\equiv |F|\}$, return to the nest with liquid food loaded at the food source. Back in the nest, the foragers transfer the food to the non-forager population, $A=\{N_{\text{foragers}}+1,N_{\text{foragers}}+2,\ldots ,N_{\text{ants}}\}$, via trophallactic interactions (Fig 1a). As food accumulates in the colony (Fig 1b–1d) it also flows between non-forager ants as they interact among themselves [17, 36].

**Fig 2:**
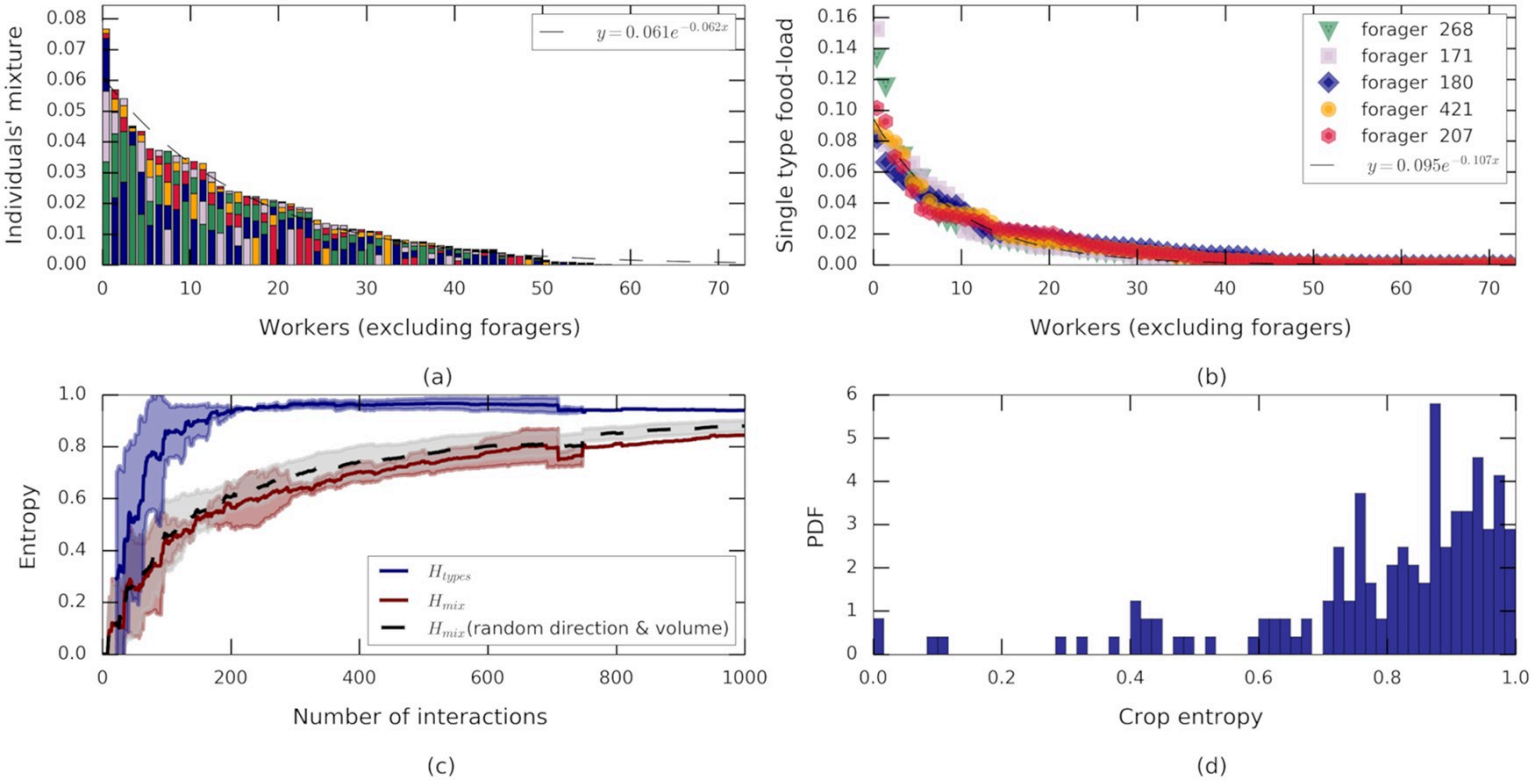
Food spread and source blending across the colony. **(a)** The amount of food held by each non-forager ant *a* at the end of the experiment, *P*(*a*), partitioned into conditional probabilities *P*(*f*|*A* = *a*) by forager origin (*f* = 268, green, *f* = 171 purple, *f* = 180 blue, *f* = 421 orange, *f* = 207 pink; vertically ordered by amounts received). Ants are ordered by the amount of food in their crop, and the dashed line is an exponential fit *y* = *ae*^−*bx*^,*a* = 0.061 ± 0.002, *b* = 0.062 ± 0.003, *R*^2^ = 0.96. For colonies B and C see S2 Fig. **(b)** The extent to which food from each forager *f* (color code as in panel a) was distributed among non-forager ants *a*: *P*(*a*|*F* = *f*). Recipient ants are ordered (per forager *f*) by amount received. Dashed curve is an exponential fit *y* = *ae*^−*bx*^, *a* = 0.095 ± 0.0013, *b* = 0.1 ± 0.002, *R*^2^ = 0.97. For colonies B and C see S2 Fig. **(c)** Mixing entropies as a function of the number of trophallactic interactions starting from the first return of a loaded forager. Entropies are normalized by log(|F|) to allow for data averaging over the three experiments. Lines are the mean over three experiments while shaded areas designate standard deviations. Depicted are the empirical entropy associated with the different proportions of food as brought in by each forager *H*_types_ (blue), the empirical mixing entropy over all non-forager ants *H*_mix_, (red) and the mixing entropy for hybrid simulations where randomized interaction volumes and transfer directions were simulated over the empirical interaction schedule (*N* = 30, shaded area depicts standard deviation of the outcomes). Discontinuities are a consequence of the variable number of interactions among the three experiments. **(d)** A histogram of normalized individual mixing entropies of all non-forager ants, $h_{\text{mix}}^{a}/\text{log}\left(|F|\right)$, at the end of the experiments (all three experiments, *N* = 203 ants).

The amount of food held in the crop of each ant as well as the amount of food passed per interaction were directly measured by combining single ant tracking with imaging of fluorescently labeled (Rhodamine B [0.08*g*/*l*]) food (Fig 1a, S1 Data) [17]. We designate the total amount of food in the crop of a non-forager ant *a* at time *t* by *n*_*a*_(*t*) and the total amount of food held by all non-forager ants by $Z\left(t\right)=\sum_{a\in A}n_{a}\left(t\right)$. During the course of an experiment, the total amount of food held by the colony grows until it reaches saturation (S1e Fig) [1, 36]. The fraction of the total food held by ant *a* by *P*_*a*_(*t*) = *n*_*a*_(*t*)/*Z*(*t*) is not uniform across colony members (Fig 2a, S2 Fig) and is restricted by variable physiological properties such as crop capacity.

As a first step towards quantifying food mixing in the ant colony we took a forager-centric approach. The idea is to track how food brought in by each forager spreads across the colony (Figs 1(b)–1(d), 2(a) and 2(b) and S1c Fig) and the degree to which these food flows may overlap and mix. Since our experiments included a single food source we implemented this approach using a computational procedure in which we define the type of each ‘food droplet’ by the index of the forager, f∈F, that had initially collected it at the food source (see ‘Food tracking’, Methods). This entails that the number of ‘food types’ in the system is taken to be equal to the number of foragers. Using the assumption that mixing of food inside the crop of an individual ant is extremely rapid when compared to the rate at which food is transferred between ants, we then tracked the trajectories of labeled food droplets as they flow through the colony (see ‘Food tracking’, Methods). This procedure allowed us to define the empirically measured probability, *P*_*a*_(*t*), described above (*P*_*a*_(*t*) can be viewed as the probability that a randomly chosen ‘food-droplet’ is found within the crop of ant *a*), and consider the inferred joint probability *P*_*f*,*a*_(*t*) = *n*_*f*,*a*_(*t*)/*Z*(*t*), which represents the probability that food, originally collected by forager *f*, is located in the crop of ant *a* at time *t*.

To quantify the degree to which different foragers contributed to the total foraging effort we calculate the total amount of food of type *f* that has accumulated in the colony up to time *t* as $P_{f}\left(t\right)=\sum_{a\in A}P_{a,f}\left(t\right)$. This probability function may be associated with an entropy, which we refer to as the *types-entropy* (*H*_types_), and which quantifies the relative abundance of the different food types (for all entropy definitions refer to SI, ‘Mathematical Framework’ and ‘Table B, S1 Text’). It is defined by:

$$\begin{array}{c} H_{\text{types}}\equiv H\left(F\right)=-\sum_{f\in F}P_{f}\text{log}\left(P_{f}\right) \end{array}$$

(we suppress the explicit notation of time from here onward). Our measurements show that *H*_types_ increases as a function of time (Fig 2c) and quickly approaches the upper bound of log(|F|). This upper bound can only be saturated if all foragers bring in equal amounts of food. As discussed below, *H*_types_ sets a limit on the total level of mixing in the colony.

The degree to which food of a given type, *i.e* food brought in by a single forager—*f*, spreads across the colony can be quantified by the conditional distribution *P*(*a*|*F* = *f*) = *P*_*f*,*a*_/*P*_*f*_. We found that the food initially collected by each and every forager reaches, practically, all members of the colony (Fig 2b). This degree of dissemination dictates overlapping food flows such that the crops of non-forager ants must hold a mixture of food of several types (Fig 1b–1d).

### Food mixing

Mixing was assessed by tracking the differently labeled food droplets as they flow, via the trophallactic network, from ant to ant. The conditional distribution *P*(*f*|*A* = *a*) = *P*_*f*,*a*_/*P*_*a*_ signifies the mixture of food-types in the crop of a specific ant *a* (Fig 2). Since each non-forager ant receives its load from multiple interactions with both foragers and non-foragers [17, 31] the food composition in her crop, *P*(*f*|*A* = *a*), contains a mixture of differently labeled ‘droplets’.

The level of blending in the crop of each individual ant, *a*, can be defined by the *crop entropy*:

$$\begin{array}{c} h_{\text{mix}}^{a}\equiv H\left(F|A=a\right)=-\sum_{f\in F}P\left(f|A=a\right)\text{log}\left[P\left(f|A=a\right)\right]. \end{array}$$

The range of individual crop entropy, $h_{\text{mix}}^{a}$, is [0,log(|F|)] where zero crop entropy indicates that all food in the ants crop originates in a single forager while log(|F|) indicates that food in the crop is equally divided among all possible food types. We find that the (non-weighted) average mixing entropy (Fig 2d) takes an intermediate value of 0.79 of the maximal possible mixing. While the actual components that mix to create the crop of each ant vary greatly (Fig 2) we find that the degree of mixing is actually quite uniform across the colony (standard deviation of 0.2·log(|F|), Fig 2d).

Mixing within the entire colony, as a whole, can be quantified by the conditional entropy, *H*(*F*|*A*). This global *mixing entropy* is defined as the weighted average over individual crop entropies, $h_{\text{mix}}^{a}$, where each ant is weighted by its relative load, *P*_*a*_ [35]:

$$\begin{array}{c} H_{\text{mix}}\equiv H\left(F|A\right)=\sum_{a\in A}P_{a}\cdot h_{\text{mix}}^{a}\;. \end{array}$$

Mixing entropy is bounded from below by zero, a value which signifies no mixing (this can happen if the food in the crop of any ant originates from a single forager only). An upper bound on mixing is obtained by the general rule *H*(*F*|*A*) ≤ *H*(*F*) (conditioning reduces entropy [35]) which, in our notation, translates into the fact that the mixing entropy is smaller or equal to the types entropy (*H*_mix_ ≤ *H*_types_). Equality signifies perfect blending and occurs only when all ants have identical crop-load compositions that exactly match the concentration-distribution of food types across the entire colony.

We find that as the number of interactions grows so does the mixing entropy, *H*_mix_ (Fig 2c, S1 Fig). However, while the crop composition of a typical ant contains food that originated from each of the foragers, the relative proportions of these food types differ from ant to ant and do not match the proportions of food types flowing into the system (Fig 2c, S1 Fig). In other words, even though the types entropy (*H*_types_) designating the partition of the total food in the colony into types, does approach the maximal bound of log(|F|), the mixing entropy (*H*_mix_) designating a similar partition on an individual level within each crop, is lower during the entire course of the experiment and reaches *H*_mix_/*H*_types_ = 0.8 ± 0.02 (mean ±std over three experiments) at the end of the experiments (Fig 2c). If the mixing entropy does eventually reach the upper bound of the types entropy the time for this to occur is very long.

To discern the causes of these intermediate mixing levels we next focus on the underlying dynamics of food exchange. In the following sections, we characterize the pairwise interactions via which food spreads through the colony and study their implications on mixing.

### The process of food transfer

The flow of food across the colony can be described by focusing on two processes: 1) The *interaction network* which is the time-ordered depiction of the pairs of ants that engage in trophallaxis. 2) The *interaction volume* which depicts food exchange during an interaction in terms of both direction and volume. Next, we briefly characterize these two components.

#### Interaction network

Quantitative characterizations of temporal networks are difficult [38, 39], and in this section, we characterize network connectivity by studying the static graph which includes all interactions. In particular, we are interested in testing whether network connectivity (or its absence) may limit mixing. More accurate descriptions that take into account the temporal structure of the network will be discussed in the next section.

**Fig 3:**
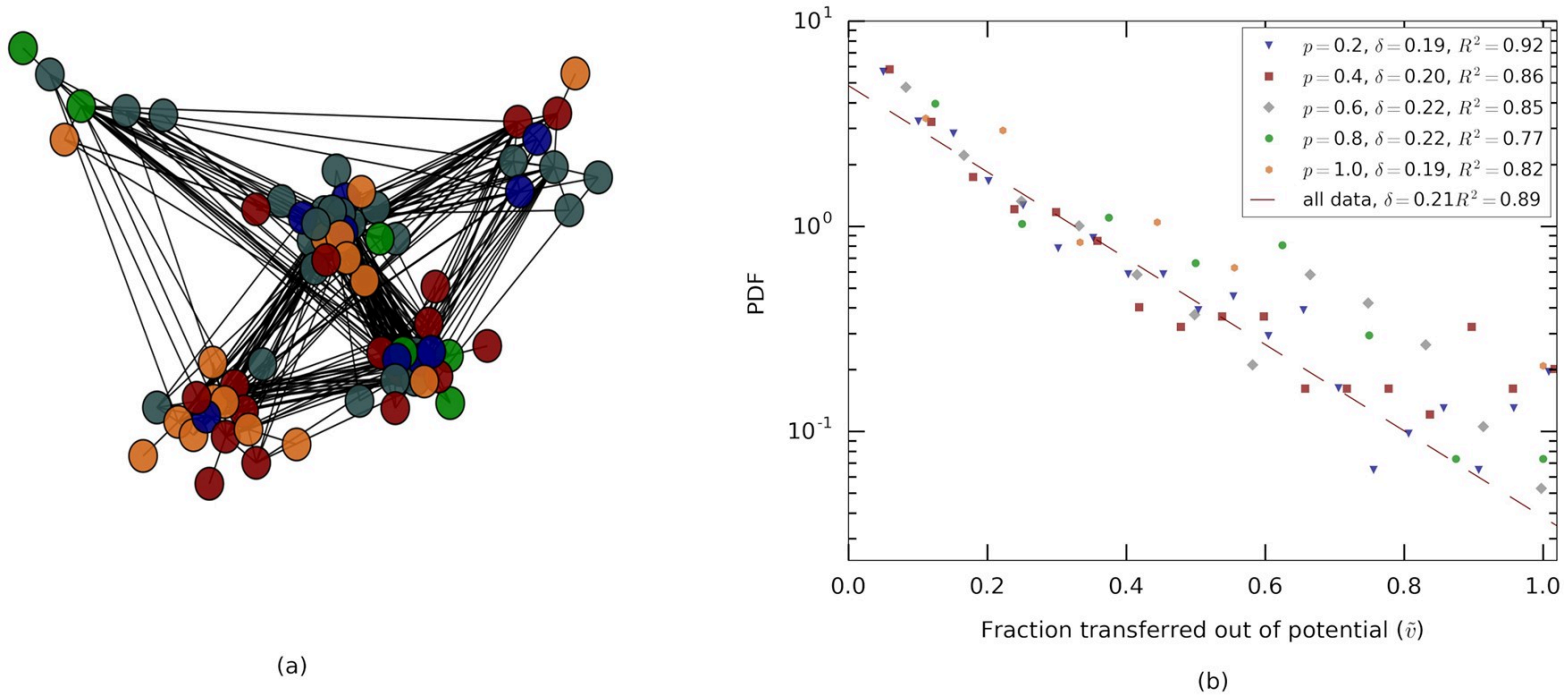
The network of pairwise interactions and the flow of food over interactions. **(a)** Visualization of the undirected trophallactic network (Colony A). Ants are represented by vertices and interactions are edges using the spring embedded layout from Networkx [37]. Nodes are colored according to maximally modular communities which, nevertheless, display low modularity with 191 intra-community and 223 inter-community links (Details: Number of communities = 5, transitivity = 0.38, modularity = 0.185, quality performance = 0.76). For colonies B and C, see S3 Fig. **(b)** Probability density as a function of the transfer ratio. Different colors relate to the maximal potential interaction *p* = *d*(1 − *r*) where *p* ∈ [0, 1] is the volume potential as determined by *d*, *r* ∈ [0, 1] donor’s and recipient’s crop load expressed as a fraction of the capacity of each ant. The distribution of interaction volumes per each transfer potential, *p*, were estimated by exponential functions $y=\frac{1}{\delta }e^{-(\tilde{v}/\delta )}$ in which the values of the parameter *δ* were determined using maximal likelihood. These values of *δ* all produce decent fits (*R*^2^ values are indicated in figure legend) and are, largely, independent of the maximal transfer potential. This implies that the ants control the fraction of volume transferred (out of the maximal possible interaction volume) rather than absolute amounts. Data depict interactions from all three experiments (*N* = 2141 interactions).

The *modularity* of a partition of a network into communities is defined as the fraction of the edges that fall within communities minus the expected fraction if the same number of edges were randomly distributed [40]. We used the ‘greedy modularity communities’ algorithm (‘Networkx’ Package for Python [37]) to search for a partition of the undirected trophallactic network which maximizes modularity. We found (Fig 3a, S3 Fig) that maximal modularity occurs for a partition into 3–5 communities, a number that is similar to previous estimates for this species [41]. However, these maximally modular divisions display low modularity (0.16 ± 0.024) in which the ratio of intra/inter edges tend to 1 (1.13 ± 0.3). We conclude that the food dissemination, most likely, is not hindered by missing links between communities.

#### Interaction direction and volume

When a forager ant interacts with a non-forager she typically acts as the donor (76 ± 16%, *N* = 713 where the error signifies interactions where direction could not be specified (S4a Fig). Moreover, whenever a forager was the recipient, the volume transferred was negligible (0.003 ± 0.008 mean±std. *N* = 240) in comparison to all other cases (0.28±0.14 mean±std. *N* = 1901).

To check for a possible choice of directionality in interactions between two non-foragers we calculated the probability that a fuller ant (as a fraction of her own capacity) passes food to the emptier one. Within the limitations of measurements we find no such effect on the direction of transfer (56 ± 17%, *N* = 1357, error bars as above, S4 Fig). These results do not change when considering absolute amounts of the ants’ food-load rather than the fraction filled (S4b Fig).

We next focus on *interaction volume*. As the crop of the ants is of finite size this limits the volume an ant can take. But the transfer is also limited by what the donor can give. Thus, we define the maximal transferable volume, *v*_max_, as the minimum between food in the donor’s crop and the free space in the receiver’s crop. The interesting finding is that, on average, the actual interaction volume, *v*, is no more than 0.26 ± 0.1, of this maximal potential regardless of how full or empty the receiver and the donor ants are (Fig 3b). Furthermore, the distribution of the fraction $\tilde{v}=v/v_{\text{max}}$ resembles an exponential:

$$\begin{array}{c} p_{\delta }\left(\tilde{v}\right)=\{\begin{array}{cc} c_{\delta }\frac{1}{\delta }e^{-v/\delta }\;, & \text{if}\;v\leq 1\;\\ 0\;, & \text{otherwise} \end{array}\; \end{array}$$

where, *c*_*δ*_ = 1/(1 − *e*^−1/*δ*^) normalizes the probability distribution.

The fact that this *food-transfer rule* acts on $\tilde{v}$ rather than on *v* itself suggests that, during interactions, ants control fractions of volume rather than absolute amounts [36]. Taking *δ* = 0.26 in the food-transfer-rule provides a good description of both the case in which the donor is a forager and the case where it is a non-forager (S4c Fig), p-value *p* = 0.98, as computed by the Kolmogorov-Smirnov statistics on a set that includes only forager to non-forager interactions versus a set that includes only interactions between two non-foragers). It was previously suggested that, in interactions between foragers and non-foragers, it is the recipient ant that controls interaction volume [36]. The fact that the interaction volume rule does not depend on the identity of the donor is therefore consistent with an assumption that the recipient ant is not aware of this identity.

### Limits on macroscopic mixing

Different aspects of the trophallactic interaction may limit food mixing in different ways. One way in which mixing levels may be reduced stems from the details of the interaction rule. As an extreme example: if the crop capacity of all ants was about equal and in any trophallactic event ants would transfer as much food as possible this would lead to pure food loads that are simply relayed between the ants and therefore minimal mixing. Decreased mixing may also be the result an interaction network which is topologically segregated into several disjoint communities with limited food flows between them (as reported for other ant species [4]). In this section, we describe hybrid simulations, which preserve some of the empirically measured data while replacing others by simulated values (for details see SI, ‘Simulations’), to separately examine the effects of the different aspects of the interaction details on overall mixing.

#### Precise interaction volumes

Food mixing within the colony can be regulated by communication and controlled interaction volumes. For example, if interacting ants can sense that they hold very different crop compositions and react by reducing the trophallactic volume, this can limit mixing on the collective scale.

We have shown that the statistics of the trophallactic interaction volumes resembles an exponential distribution (Fig 3b). In line with the above reasoning, this distribution might stem from precisely controlled interactions governed by internal parameters which we did not measure. However, an exponential distribution may also be the result of a random process where Poisson-like dynamics govern the termination of an interaction. A similar ambiguity holds for our measurements regarding the directionality of the interaction. As external observers, we have no way of telling random from non-random in this case. Nevertheless, we can assess the effect of possible randomness on the process of mixing.

To this end, we ran hybrid simulations in which ants interact according to the empirically measured network but in which interaction directions are chosen uniformly at random and interaction volumes are stochastically generated according to the empirical exponential food-transfer rule distribution (Fig 3b). We find that these hybrid simulations exhibit limited mixing levels that are similar to the measured ones (Fig 2c). In other words, the dynamics of food mixing does not suggest that the interacting ants use intricate communication, controlled directionality, or accurate interaction volumes.

#### Maximally mixing interaction rule

The clustering analysis presented above suggests that reduced levels of mixing are not a consequence of the network structure. However, this analysis was performed on a fixed interaction network that does not capture the temporal order at which interactions occurred.

To more accurately test whether interaction network properties limit mixing we ran hybrid simulations in which we kept the empirically measured interaction schedule including ant identities but replaced the measured interaction volumes by maximally mixing interactions: At each interaction, each ant gives half of her own crop to her trophallactic mate: Both ants leave the interaction with identical crop loads in terms of both volume and composition. Note that these interactions do not necessarily respect the empirically measured limited physical volume of each ants’ crop.

**Fig 4:**
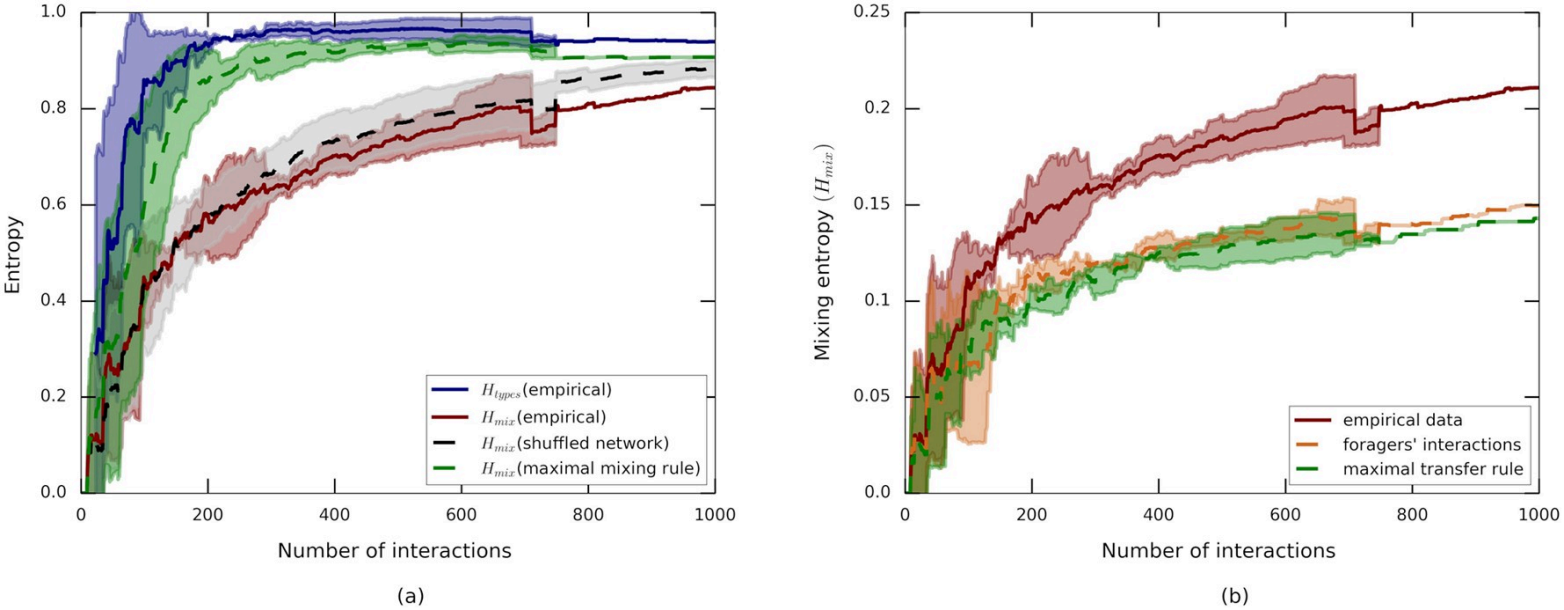
Mixing as a function of the number of interactions. Plots compare empirical data and hybrid simulations. Entropies are normalized by log(|F|), solid lines show the empirical mean over the three experiments, dashed lines represent means over hybrid simulations. Shaded areas depict standard deviations. **(a)** The mixing entropy, *H*_mix_, in simulations with maximally mixing interactions applied over the empirically measured network (green curve) nearly saturates the empirically assessed upper bound *H*_types_ (blue curve). Mixing entropy, *H*_mix_, in simulations where the empirically derived interaction rule is applied over maximally mixing interaction networks shows a limited rise which compares with empirical mixing rates (red curve). **(b)** Hybrid simulations of two extreme interaction rules preserving the empirically measured interaction schedule. The orange curve shows *H*_mix_ using only the transfers between foragers and non-foragers (*i.e*., all non-forager to non-forager interactions were set to zero). The green curve depicts *H*_mix_ where every transfer is assumed to be at its maximal possible volume. These rules lead to mixing levels that are lower than those measured experimentally (red curve). Discontinuities in the plots are a consequence of the variable number of interactions among the three experiments.

We find that the simulation curve of this ‘maximal-mixing’ rule exceeds the experimental data and leads to near maximal (*i.e*., *H*_types_) mixing levels (Fig 4a). In other words, the connectivity and temporal structure of the trophallactic network can support maximal homogenization and are therefore not limiting factors on the mixing dynamics. This agrees with the observation that the static interaction network shows no clear community structure (Fig 3a, S3 Fig).

#### Maximally mixing interaction network

To test whether the interaction rule limits mixing we replaced the empirical interaction network with randomly generated encounter patterns that are non-structured and do not inhibit mixing. These random networks were obtained by shuffling all individual identities in the measured interaction schedule table. This procedure allows us to preserve the number of interactions per individual while replacing the interaction network with one which is maximally mixing.

Since this shuffling process yields interactions that did not actually occur we used the empirically measured food-transfer-rule to simulate random interactions (in both directions and volume) over the simulated networks. As shown in the previous sections and in Fig 2c, this replacement is not expected to have implications on the global mixing process.

We find that simulated mixing over shuffled networks did not show any statistically significant deviation from the empirically measured mixing process (Fig 4a). We therefore conclude that the statistical properties of the ants’ interaction rules, which respect the physical capacity of the crop, will limit food mixing within the colony.

#### Extreme interaction rules

Our observations thus far raise the interesting possibility that the value of *δ*, which determines the typical fraction of the maximal potential transfer that is actually realized, is a regulator of mixing. To explore this direction, we start by looking at two extreme cases: *δ* = 0 (applied only for interactions with non-forager donors) and *δ* ≫ 1 (applied over all interactions).

We ran a hybrid simulation in which secondary interactions were turned off by setting *δ* = 0 if the donor was a non-forager. All forager to non-forager interactions were maintained at their empirical values (corresponding to *δ* = 0.26). Comparing this simulation to a simulation in which all interactions are allowed (*i*.*e*., *δ* does not depend on the identity of the donor) provided us with a means of quantifying the role of secondary interactions in the mixing process.

We find that this manipulation reduces mixing levels and conclude that secondary interactions between pairs of non-foragers indeed contribute to food mixing within the colony (Fig 4b).

The interaction dictated by *δ* ≫ 1 is that of maximal transfer. Here, the empirically determined donor-ant was simulated to transfer a volume of *v*_max_ (as defined above) which is the maximal possible interaction size given the volume constraints. Similar to the *δ* = 0 case, the maximal transfer rule shows a reduced level of mixing (Fig 4b). Note that mixing levels do not go to zero. The reason is that the crop capacity is not equal across all the workers so when food is transferred it is also divided.

To conclude, mixing levels indeed depend on the degree, *δ*, to which ants fill up during interaction. This dependence in non-monotone and displays reduced levels of mixing when the parameter *δ* is either too high or too low.

### Trade-off between mixing and accumulation rates

Finite crop size naturally impacts an ant’s ability to mix food. Mixture composition can significantly change only if an ant receives a large enough portion relative to her present load. Therefore, as ants become more satiated, their free storage space (*i.e*., the difference between her capacity and her current load) becomes smaller and the ability to mix (the potential mixing rate) declines. Consequentially, a fast accumulation rate might interfere with the mixing process.

As implied by the empirical interaction rule, in a receiving interaction, an ant is provided with a random volume of food that follows an exponential distribution, with an average that is proportional to her free storage space. This means that on average, an ant receives food in a series of decreasing volumes with a parameter *δ*. The parameter *δ* can thus be expected to have opposite effects on the accumulation and mixing of the food: the larger the value of *δ* the higher the accumulation rate and the lower the mixing rate (and *vice versa*).

We used a simple model to explore the possible trade-offs between the rate at which food accumulates within the colony and the extent to which it is mixed. For simplicity, the model assumes that all ants have the same capacity, that foragers and non-foragers use the same $\tilde{\delta }$ (in a deterministic version of the original food-transfer rule, see SI, ‘Simulations’) and that interactions occur randomly. Furthermore, for the purpose of the model, we defined the amount of food held by a forager at time *t* = 0 to equal the total amount of food she collects at the food source during the entire course of the experiment. This definition sets the amount of food summed over all colony members, *M*, as a quantity that is conserved over time. Considering the entire colony we now define the probability ${\tilde{P}}_{a}=n_{a}\left(t\right)/M$ as the fraction of total amount of food held by any ant, forager or non-forager.

Using these definitions entails that at *t* = 0 all food is held by the foragers being, therefore, completely non-mixed while at later times, as food flows into the colony, it mixes within the crops of non-forager ants. This interplay between food accumulation and food mixing can be captured by considering the mixing entropy over all ants in the colony:

$$\begin{array}{c} H_{\text{mix}}^{\text{overall}}=\sum_{a\in A\cup F}{\tilde{P}}_{a}h_{\text{mix}}^{a} \end{array}$$

Note that since foragers receive almost no food from other workers (see above) we can approximate *P*(*f*′|*a* = *f*) ≈ 1 for *f*^′^ = *f* and zero otherwise. This means that $h_{\text{mix}}^{f}=0$ for f∈F and leads to a second representation of $H_{\text{mix}}^{\text{overall}}$ (see SI, ‘Trade-off model’):

$$\begin{array}{c} H_{\text{mix}}^{\text{overall}}=P_{\text{colony}}\cdot H_{\text{mix}} \end{array}$$

where $P_{\text{colony}}=\sum_{a\in A}{\tilde{P}}_{a}$ is the colony’s satiation level which starts off at 0 and saturates at 1 as food flows into the system [17] and *H*_mix_ is the mixing entropy over all non-forager ants, as defined above. This representation neatly separates the dissemination behavior into a component which quantifies the extent at which food is accumulated and a second component which quantifies the extent at which it is mixed.

**Fig 5:**
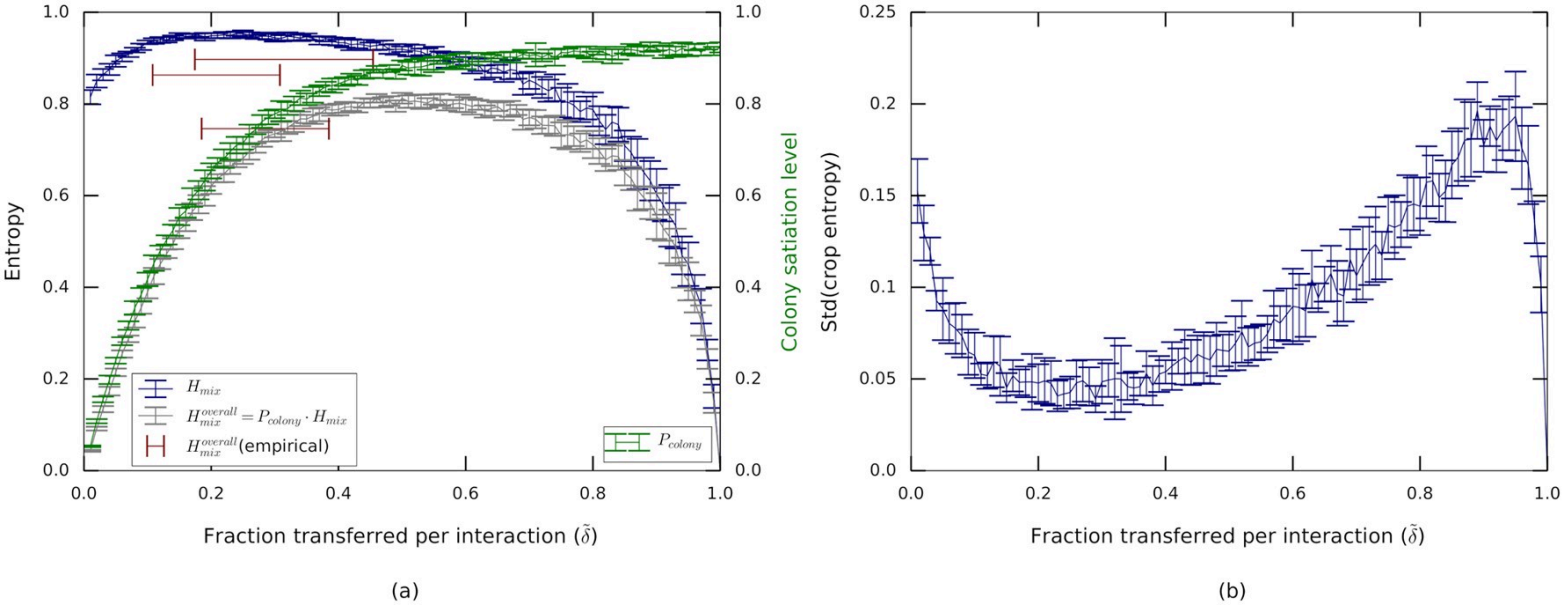
Trade-off between fast dissemination and efficient mixing described by the $\tilde{\delta }$ model. **(a)** While the colony state (*P*_colony_, green) rises with the fraction $\tilde{\delta }$ of transferred volume, mixing levels among non-foragers (which we call *H*_mix_) decrease. The mixing levels over all ants in the colony (including the foragers) is the product of these two functions, $H_{\text{mix}}^{\text{overall}}=P_{\text{colony}}\cdot H_{\text{mix}}$ and displays a broad maximum which spans all non-extreme values of $\tilde{\delta }$. Curves can be compared to the empirically measured values (red bars) of the three experiments. **(b)** Standard deviation of individual mixing entropies $h_{\text{mix}}^{a}$ across all ants. Standard deviations were calculated for 30 model runs. The plot depicts the mean and standard deviation of this value.

We simulated an approximation to this model (see SI, ‘Simulations’) to study the relative effects of these terms as a function of the parameter $\tilde{\delta }$. The interactions of the simulations approximate the empirical data by keeping the average number of interactions per ant and the ratio between forager to non-forager and non-forager to non-forager interactions. As may be expected, larger values of the parameter $\tilde{\delta }$ lead to larger transfer of food into the colony (*P*_colony_ indicated by the green line in Fig 5a). However, due to the finite capacity of an ant’s crop, larger values of $\tilde{\delta }$ also hamper mixing among non-foragers (*H*_mix_ indicated by the blue line in Fig 5a). The compromise between these two factors is captured by their product, the total mixing entropy $H_{\text{mix}}^{\text{overall}}$. This entropy exhibits a maximum for an intermediate value of $\tilde{\delta }$.

These results demonstrate a robust process: as long $\tilde{\delta }$ does not approach the extremes, both the mixing and the accumulation are comparable for a given number of interactions (Fig 5a). Surprisingly, even though higher $\tilde{\delta }$ will result in a higher accumulation rate, the ants seem to function at smaller $\tilde{\delta }$ values (red bars in Fig 5a). A potential benefit of smaller $\tilde{\delta }$ values is the maintenance of similar mixing levels across all ants in the colony (Fig 5b). This stands in agreement with our empirical evaluation of the variance in mixing levels across the colony (Fig 2d).

## Discussion

It is well known that social insects manage their nutrient resources on the collective level and also on finer scales because the colony channels foods with different nutritional composition to different sub-populations. In this paper, we put forward the idea that this intricate regulation relates to the interplay between food dissemination and food mixing within the colony. High levels of dissemination are important as they ensure that any food type is available to any ant. On the other hand, high dissemination induces mixing and this reduces the required variety of nutritional choices within the colony.

A main finding of this work is that, despite repeated trophallactic interactions between the ants, food in the colony does not become evenly mixed. Quantifying mixing using entropy measures we showed that, compared to what was theoretically possible, mixing is slow to rise and levels up at around 80% of the full mixing potential. The logarithms in the definition of entropy make the significance of this number difficult to assess. For intuition, in the case of only two food sources, the maximal mixing entropy (1 bit) corresponds to each crop holding equal parts of the food sources (1: 1) while 80% of this (0.8 bits) corresponds to, a far from perfect, 3: 1 partition of food sources. This imperfect mixing offers the possibility for receiving ants to choose from a wide spectrum of nutritional compositions when the donors provide different blends. Such choices can allow ants within the nest to reach their nutritional target using feeding schemes similar to those described by the geometrical framework for food foraging [42].

We further explored the mechanisms that allow for intermediate levels of food blending. Using hybrid simulations, we found that the interaction network over which food flows does not pose any limits on mixing levels. Rather, it is the interaction rule employed by the ants that regulates the extent to which food blends. This is reminiscent of several examples in which cellular pathways with identical architecture can achieve starkly different regulatory behaviors depending on actual rate coefficients [43, 44]. Regulation by interaction rules rather than by meeting patterns is an intriguing possibility for social insects in which different collective functions often reside over very similar interaction networks [29]. For example, while proximity is required for both food sharing and disease transmission [45] different interaction rules may ensure that one of these is enhanced while the other is suppressed.

Quantifying a large number of trophallactic interactions, we directly measured the food-transfer rule (see also [36]) used by the ants. We stress several important aspects of this rule. First, the rule respects the physical limits on crop size of the ants. Broadly speaking, this limit along with the fact that ants receive a substantial fraction of their free crop space per each interaction imply that an ant may become relatively full following her first few interactions. Thus, an ant’s mixing entropy is, to a large extent, determined by a small number of large events. Since these events are random both in order and in volume it is likely that mixing entropies will not saturate their maximal upper limit (see SI, ‘Entropy by largest events’, S5 Fig). Second, we show that the interaction rule is most likely stochastic in nature and, therefore, does not entail any strong requirements on ant cognition or communication. Finally, the fact that in trophallactic interactions the recipients fill only partially (Fig 3) is in agreement with a model in which, similar to animals foraging in the environment, ants in the nest regulate their nutritional income by feeding off of multiple partners each with a different mixture of the available ‘food types’.

We explored the interplay between food dissemination and mixing using a simple model of food flow that is based on our empirical observations. We find that the intermediate levels of mixing, as measured, can viewed as a compromise between the requirements to quickly unload incoming food and the requirement to disseminate different food types to all parts of the colony. We show that this process is robust over a wide range of *δ* values and that the actual measured parameter ensures that all ants in the colony are equally well mixed (although each holds a different particular mixture).

Finally, we wish to highlight the limitations of this study. Due to current technological availability, this work was performed using a single food source labeled by a single dye. The ants may behave differently in terms of both interaction network and food-transfer-rule when several food sources with different nutritional values are available [4]. For example, ants may modulate the amount of food they receive in a trophallactic interaction according to its nutritional value. Such modulation, which can be captured in an extension of our current model, can allow the ants to differentially regulate the flow of different nutritional types across the colony. Further, our artificial setup contained a single chamber nest. More realistic, multi-chambered, nest structure may induce interaction networks that are more clustered than the one measured here. This may hold important consequences for nutrition dissemination. Last, is our choice to measure mixing by labeling food types by foragers. While arbitrary, this is a reasonable choice since, as we have shown, foragers are responsible for a large part of the mixing (Fig 4b). Taking all these limitations into account we view our findings as a baseline to which future results, where multiple food sources are provided and tracked may be compared to.

Overall, our finding that the interaction rule takes precedence over the interaction schedule manifests both the robustness of collective processes within the ant colony and the large extent to which individual behaviors may modulate global outcomes.

## Materials and methods

For a more comprehensive methods section please refer to the SI and [17, 36]. Our experiments were conducted on lab colonies of *Camponotus sanctus* which included 50-100 workers, reared from single queens that were collected during nuptial flights in Neve Shalom and Rehovot, Israel. ‘Table A, S1 Text’ contains further details on each experimental colony.

### Experimental setup

The experimental setup consisted of an IR-sheltered artificial nest chamber (^~^100 cm^2^), neighboring an open area which served as a yard. The setup was recorded by two cameras (details in [17]): the top camera images were used to extract ant identities, coordinates and orientations using the BugTag software (Robiotec, Israel). The bottom camera images were used to detect fluorescent-labelled, using the openCV library in Python. Combining the information from both images, we associated between the identity of an ant and her appropriate fluorescent image. Thus, for each experiment a database was obtained, which included for every frame the coordinates, orientation, and measured fluorescence (in arbitrary units of pixel intensity) of each identified ant.

### Food tracking

The experimental trophallactic network includes a time-ordered pairwise-interaction schedule, and the volume of liquids that one ant passed (received) to (from) the other (see S1 Data). Food is tracked from the moment it is acquired by a forger from the food source. We associate this volume (food ‘droplets’) with the forager’s barcode identity (‘type’), and continue tracing these droplets as they split between the ants according to the interaction schedule. To do this, we assume that in each interaction the receiver ant receives a fraction of the donor’s food in which the food type distribution is identical to that of the donor. In other words the number, $n_{\text{receiver}}^{\prime }\left(f\right)$ of food droplets of type *f* in the receiver’s crop, following an interaction, is given by:

$$\begin{array}{c} n_{\text{receiver}}^{\prime }\left(f\right)=n_{\text{receiver}}\left(f\right)+\frac{v}{V_{\text{donor}}}\cdot n_{\text{donor}}\left(f\right) \end{array}$$

where *n*_*x*_(*f*) is the number of food droplets of type *f* in an ant’s crop before the interaction and $\frac{v}{V_{\text{donor}}}$ is the fraction of the donors crop content that is transmitted during the interaction. The updated distribution of food types in the receivers’ crop following the interaction is given by:

$$\begin{array}{c} P\left(f|A=receiver\right)=\frac{n_{\text{receiver}}^{\prime }\left(f\right)}{\sum_{\phi \in F}n_{\text{receiver}}^{\prime }\left(\phi \right)} \end{array}$$

Note that the number of food droplets per milliliter of food is arbitrary and cancels out in this calculation. The interaction networks for all three colonies including interacting ants’ identities, time, and interaction volumes can be found in the accompanying file ‘S1 Data’.

## Supporting information

S1 FigColony entropy.Related to Fig 2. Each color stands for a different experiment (blue: colony A (also shown in the main text), green: colony B, grey: colony C). The upper edge of each colored area represents the entropy as calculated from the experimental data. The lower edge (dashed line) depicts the results of a hybrid simulation in which interaction between non-forager workers are excluded. **(a)** Overall dissemination entropy normalized by log(*N*_ants_)). **(b)** Mixing entropy normalized by log(|F|)). **(c)** Foragers’ dissemination entropy normalized by log(*N*_ants_)). **(d)** Sources entropy normalized by log(|F|)). **(e)** Colony state—normalized total amount of food in the colony as a function of number of interactions.(TIF)

S2 Fig Food distribution. Relates to Fig 2 in the main text (’Food spread and source blending across the colony’). **(a and b)** *P*(*a*|*F* = *f*): y-axis represents the fraction of food of source *f* in ant with index x. Index x (x -axis) is sorted from the largest to the smallest, for colonies B and C respectively. Each color stand for a different forager (*source*). **Dashed line (a)**- fit: *y* = *ae*^−*bx*^, *a* = 0.086±0.005, *b* = 0.089 ± 0.008, *R*^2^ = 0.87.**(b)** Colony C: *P*(*a*|*F* = *f*) **Dashed line (b)**- fit: *y* = *ae*^−*bx*^, *a* = 0.082 ± 0.001, *b* = 0.085 ± 0.002, *R*^2^ = 0.97. Figs. a and b relate to colonies C and B respectively. **(c and d)** *P*(*f*|*A* = *a*): y-axis represents the fraction of food of source *f* in ant a. Ants (x -axis) are sorted from the largest to the smallest according to their crop load at the end of the experiment, for colonies B and C respectively. Each color stand for a different forager (*source*) and sorted within each bar according to the fraction of the individual crop load. **Dashed line (c)**- fit: *y* = *ae*^−*bx*^, *a* = 0.09 ± 0.0035, *b* = 0.1 ± 0.0055, *R*^2^ = 0.81. **Dashed line (d)**- fit: *y* = *ae*^−*bx*^, *a* = 0.09 ± 0.0015, *b* = 0.093 ± 0.002, *R*^2^ = 0.93. Figs. c and d relate to colonies C and B respectively.
(TIF)

S3 Fig Trophallactic network. Relates to Fig 3. Visualization of the undirected trophallactic network in which ants are the vertexes (circles) and interactions are edges (black lines), laid out with the spring embedded layout from Networkx [37] according to communities (colors). (a)—Colony C: The abundance of inter communities edges is high (177 inter edges and 275 intra edges) and the division to community does not capture the structure of the topology (number of communities = 3, transitivity = 0.537, modularity = 0.128, quality performance = 0.65). (b)—Colony B:. This maximal modularity partition shows the same number of intra-community edges (n = 170) as inter-community edges (n = 173) suggesting that division into communities does not capture the n topology of this network (number of communities = 4, transitivity = 0.4, modularity = 0.174, quality performance = 0.7).
(TIF)

S4 Fig Interaction rules. Relates to Fig 3 (’The network of pairwise interactions and the flow of food across an interaction’). **(a)** The direction of trophallactic interaction. Grey bars—the fraction of interactions in which the donor was more full for three different subgroups: all-interactions (*N* = 2141, 0.59 ± 0.14), non-forager—non-forager (*N* = 1357, 0.56 ± 0.17) forager -non-forager (*N* = 713, 0.64 ± 0.16). Error bar stand for the events in which the transferred volume was below the detection error. The tendency to be higher than 0.5 may be explained by the cases in which the recipient was empty, in this case food can flow in one direction only. Blue bar-fraction of events in which the donor was a forager out of all interactions that include forager and a non-forager worker (*N* = 713, value = 0.76). Green bar- fraction of events in which the direction could not be determine, (either because nothing was transferred or due to measurement error) out of all interactions that include forager and a non-forager worker (*N* = 713, value = 0.16) Yellow bar—fraction of events in which the recipient was a forager out of all interactions that include forager and a non-forager worker -(*N* = 713, value = 0.07). **(b)** Similar to **a** but here the grey bars signify the fraction of interactions in which the donor’ crop load was greater than the recipient’s load: All-interactions (*N* = 2141, 0.59 ± 0.14), non-forager—non-forager (*N* = 1357, 0.58 ± 0.17), forager -non-forager (*N* = 713, 0.61 + 0.16). **(c)** *δ* rule for foragers and non-foragers: Blue—interaction between foragers and non-forager workers (*N* = 713), Green- interactions between non-forager workers (*N* = 1357). The two cases show no obvious difference (Kolmogorov-Smirnov statistic on 2 samples: KS statistic = 0.067, pvalue = 0.07). **(d-f)** **2d trophallactic direction plots**. Data included all interactions from the three experiments (*N* = 2141), and was binned according to the trophallactic-pair level of satiety as a fraction of the capacity of each ant (x-axis the ant with the lower satiety level, y-axis the ant with the higher satiety level). Colors indicate: **(d)** Fraction of interaction in which the donor ant was fuller. **(e)** Number of events. **(f)** Fraction of near-zero volume events in which direction could not be determined.
(TIF)

S5 Fig Crop entropy and the entropy generated by the largest events. Relates to Fig 5. The number of largest events is chosen as the number of foragers in the experiment. **a)** PDF of crop entropy (grey) and the entropy generated by the largest events (yellow). Kolmogorov-Smirnov statistic on 2 samples: KS statistic = 0.15, pvalue = 0.13. **b)** PDF of crop entropy (grey) and the entropy generated by the sequence (red) *m*_1_, *m*_2_, ..*M*_*n*_, where *m*_*j*_ = *δ*(1 − *δ*)^*j*−1^, *δ* = ‘scale’ = 0.196 and *n* = number of foragers. Kolmogorov-Smirnov statistic on 2 samples: KS statistic = 0.01, pvalue = 0.57.
(TIF)

S1 Movie Food dissemination in ant colony. Relates to Fig 1(b)–1(d). Visualization of the process of food dissemination and mixing based on the experimental data. The process begins with foragers, who feed directly at the food source. Food collected by a forager is labeled according to forager identity: forager-268: green, forager-171: orchid, forager-207: red, forager-421: Yellow and forager-180: Blue. Colored blobs overlaid on the movie depict the computationally determined amount of food each ant carries. Each blob is composed of colored transparent layers. The long axis of the ellipse depicting each color is proportional to the amount of food held by the ant which is associated with the forager corresponding to this color (*i.e*., food that was originally collected at the food source by this forager.).
(MP4)

S1 Text Supplemental information. Detailed methods and mathematical background.
(PDF)

S1 Data Interaction data file. Interaction data for three different colonies. Includes ant IDs, interaction times, and interaction sizes.
(XLSX)
