# Supplementary figures and images for: Colony entropy—Allocation of goods in ant colonies
Source: PLoS Comput Biol. 2019 Aug 5;15(8):e1006925. doi: 10.1371/journal.pcbi.1006925 (PMC6681937; doi:10.1371/journal.pcbi.1006925)

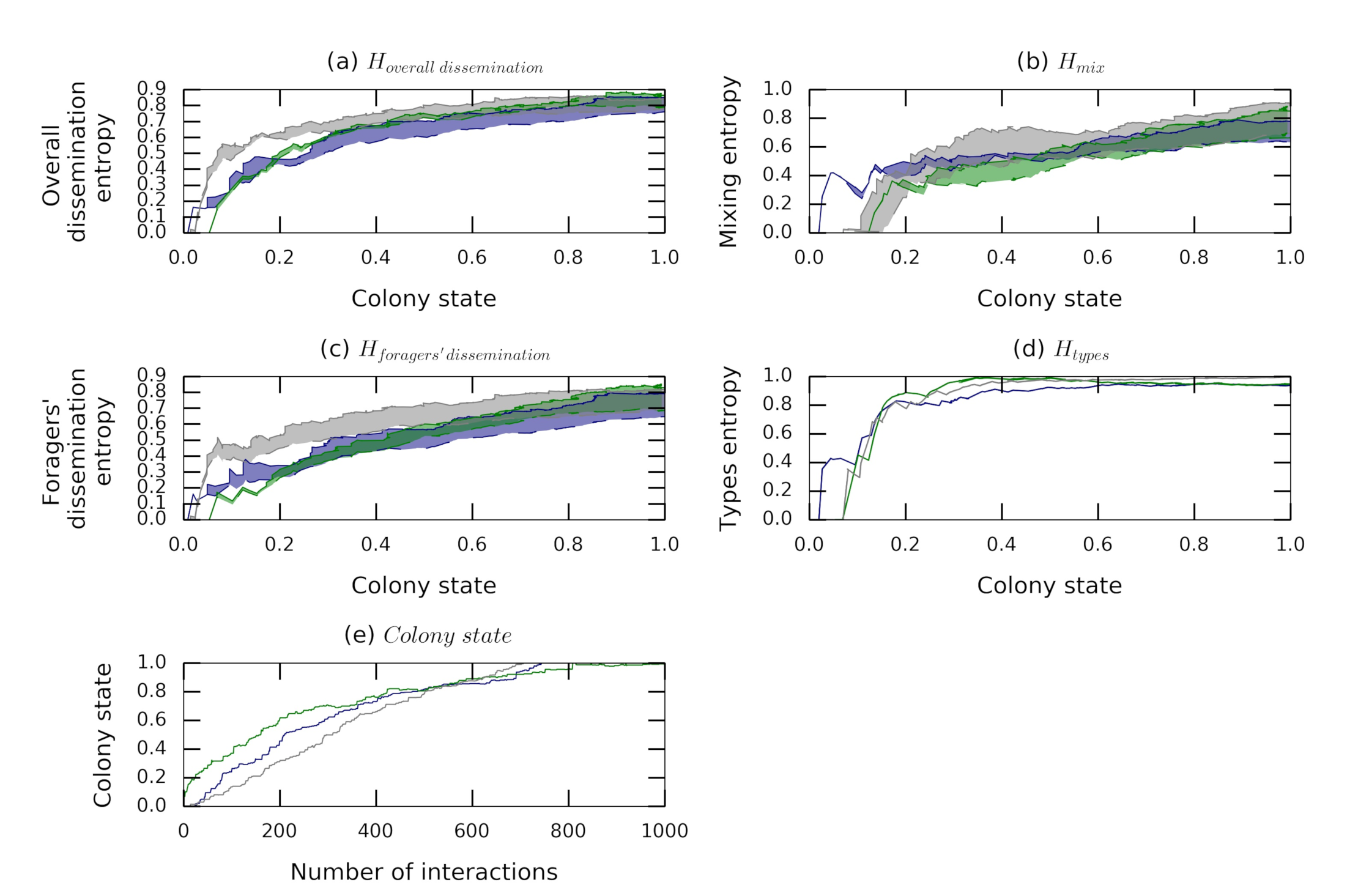

Supplement: S1 Fig — Related to Fig 2. Each color stands for a different experiment (blue: colony A (also shown in the main text), green: colony B, grey: colony C). The upper edge of each colored area represents the entropy as calculated from the experimental data. The lower edge (dashed line) depicts the results of a hybrid simulation in which interaction between non-forager workers are excluded. (a) Overall dissemination entropy normalized by log(Nants)). (b) Mixing entropy normalized by log(|F|)). (c) Foragers’ dissemination entropy normalized by log(Nants)). (d) Sources entropy normalized by log(|F|)). (e) Colony state—normalized total amount of food in the colony as a function of number of interactions. (TIF) [file pcbi.1006925.s001.tif]

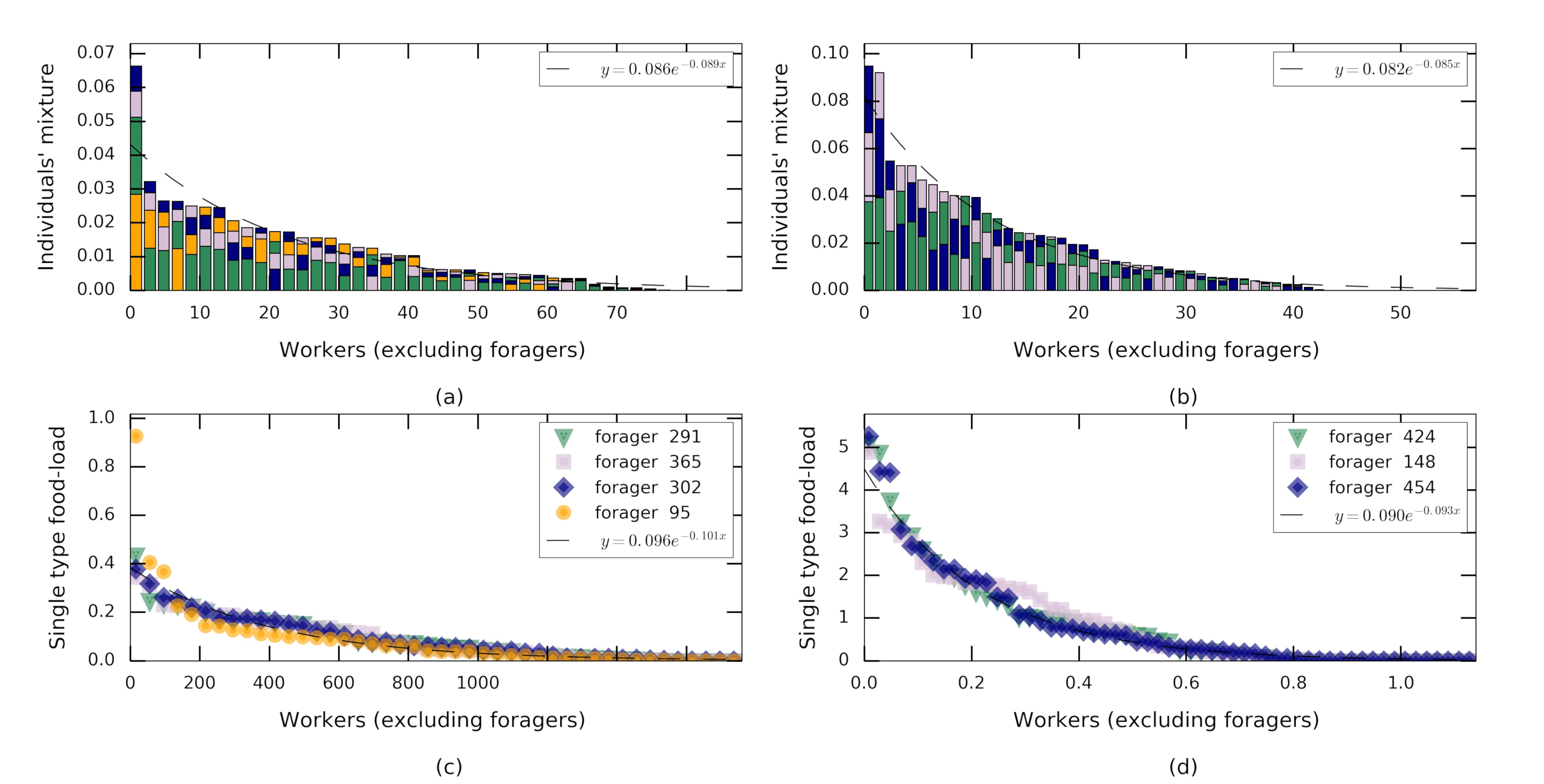

Supplement: S2 Fig — Relates to Fig 2 in the main text (’Food spread and source blending across the colony’). (a and b) P(a|F = f): y-axis represents the fraction of food of source f in ant with index x. Index x (x -axis) is sorted from the largest to the smallest, for colonies B and C respectively. Each color stand for a different forager (source). Dashed line (a)- fit: y = ae−bx, a = 0.086±0.005, b = 0.089 ± 0.008, R2 = 0.87.(b) Colony C: P(a|F = f) Dashed line (b)- fit: y = ae−bx, a = 0.082 ± 0.001, b = 0.085 ± 0.002, R2 = 0.97. Figs. a and b relate to colonies C and B respectively. (c and d) P(f|A = a): y-axis represents the fraction of food of source f in ant a. Ants (x -axis) are sorted from the largest to the smallest according to their crop load at the end of the experiment, for colonies B and C respectively. Each color stand for a different forager (source) and sorted within each bar according to the fraction of the individual crop load. Dashed line (c)- fit: y = ae−bx, a = 0.09 ± 0.0035, b = 0.1 ± 0.0055, R2 = 0.81. Dashed line (d)- fit: y = ae−bx, a = 0.09 ± 0.0015, b = 0.093 ± 0.002, R2 = 0.93. Figs. c and d relate to colonies C and B respectively. (TIF) [file pcbi.1006925.s002.tif]

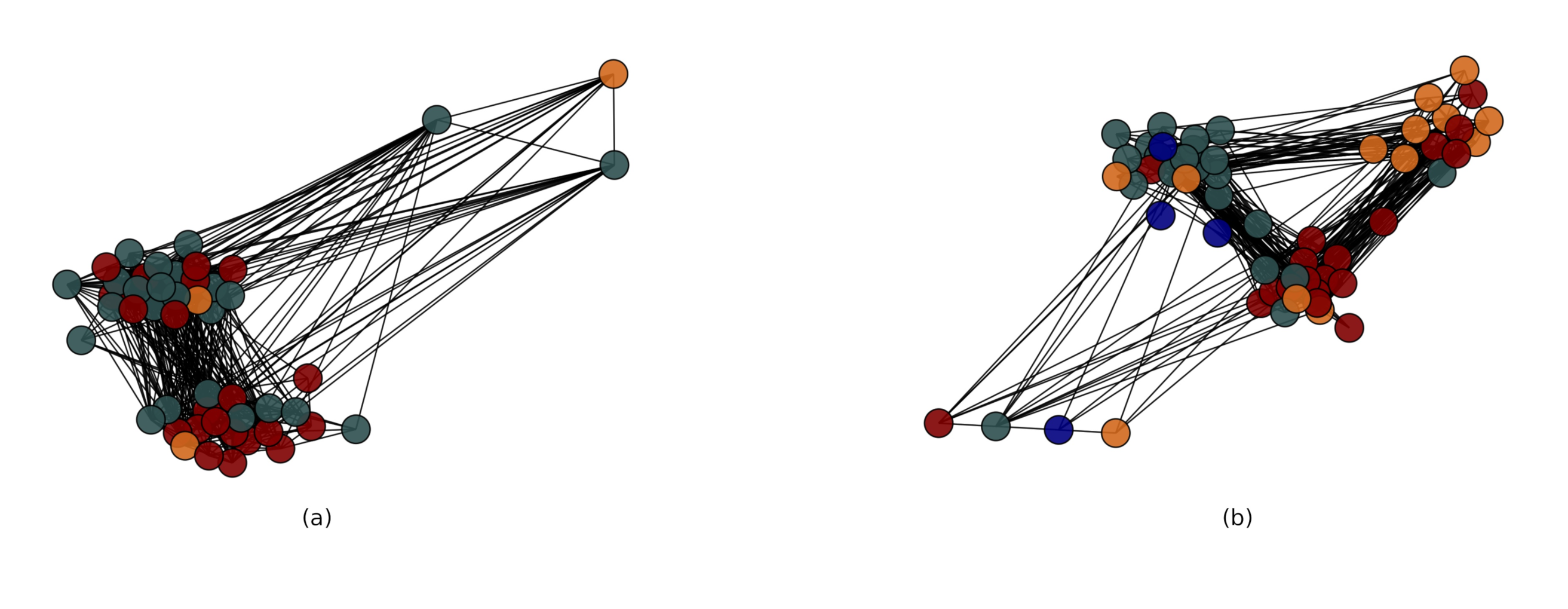

Supplement: S3 Fig — Relates to Fig 3. Visualization of the undirected trophallactic network in which ants are the vertexes (circles) and interactions are edges (black lines), laid out with the spring embedded layout from Networkx [37] according to communities (colors). (a)—Colony C: The abundance of inter communities edges is high (177 inter edges and 275 intra edges) and the division to community does not capture the structure of the topology (number of communities = 3, transitivity = 0.537, modularity = 0.128, quality performance = 0.65). (b)—Colony B:. This maximal modularity partition shows the same number of intra-community edges (n = 170) as inter-community edges (n = 173) suggesting that division into communities does not capture the n topology of this network (number of communities = 4, transitivity = 0.4, modularity = 0.174, quality performance = 0.7). (TIF) [file pcbi.1006925.s003.tif]

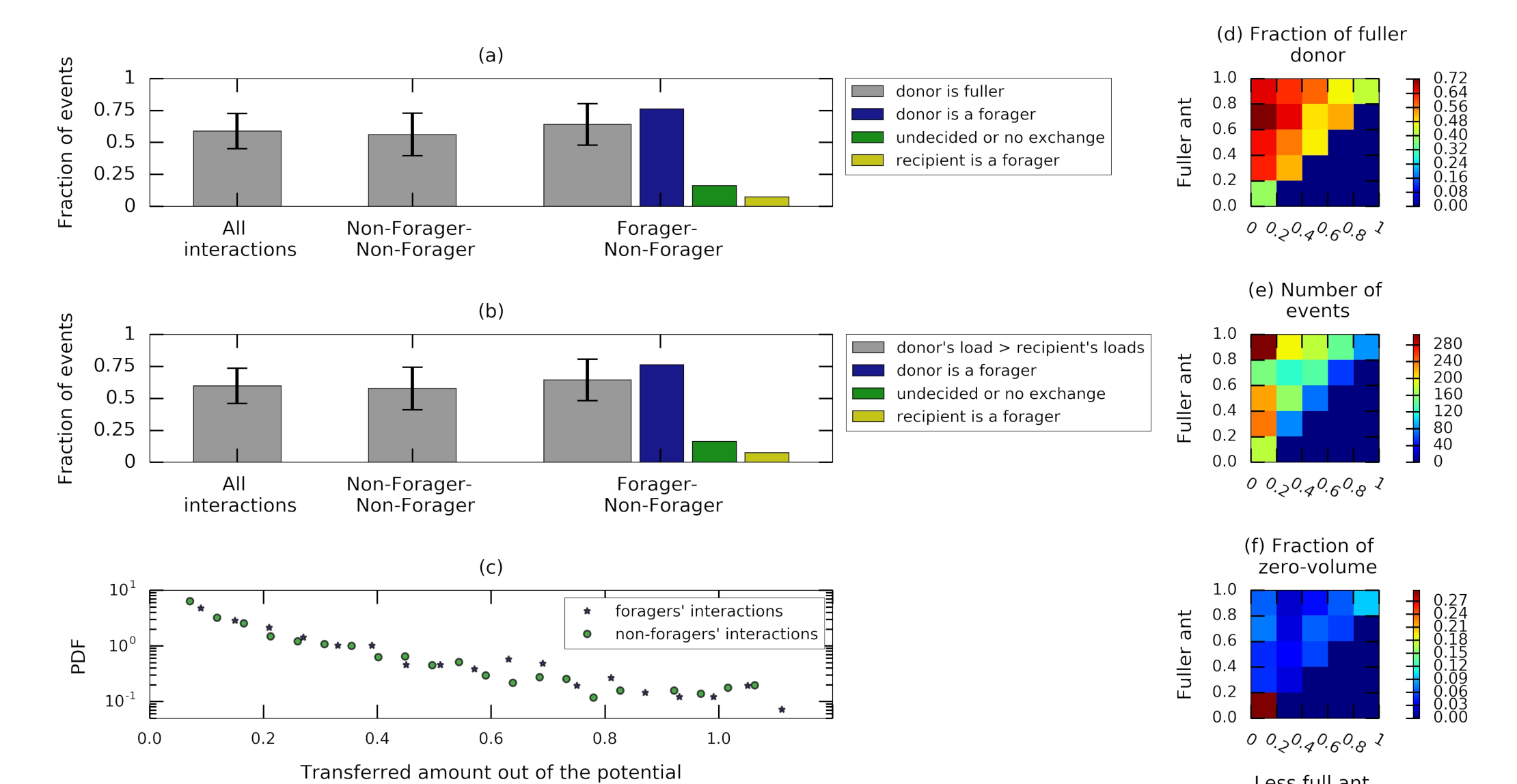

Supplement: S4 Fig — Relates to Fig 3 (’The network of pairwise interactions and the flow of food across an interaction’). (a) The direction of trophallactic interaction. Grey bars—the fraction of interactions in which the donor was more full for three different subgroups: all-interactions (N = 2141, 0.59 ± 0.14), non-forager—non-forager (N = 1357, 0.56 ± 0.17) forager -non-forager (N = 713, 0.64 ± 0.16). Error bar stand for the events in which the transferred volume was below the detection error. The tendency to be higher than 0.5 may be explained by the cases in which the recipient was empty, in this case food can flow in one direction only. Blue bar-fraction of events in which the donor was a forager out of all interactions that include forager and a non-forager worker (N = 713, value = 0.76). Green bar- fraction of events in which the direction could not be determine, (either because nothing was transferred or due to measurement error) out of all interactions that include forager and a non-forager worker (N = 713, value = 0.16) Yellow bar—fraction of events in which the recipient was a forager out of all interactions that include forager and a non-forager worker -(N = 713, value = 0.07). (b) Similar to a but here the grey bars signify the fraction of interactions in which the donor’ crop load was greater than the recipient’s load: All-interactions (N = 2141, 0.59 ± 0.14), non-forager—non-forager (N = 1357, 0.58 ± 0.17), forager -non-forager (N = 713, 0.61 + 0.16). (c) δ rule for foragers and non-foragers: Blue—interaction between foragers and non-forager workers (N = 713), Green- interactions between non-forager workers (N = 1357). The two cases show no obvious difference (Kolmogorov-Smirnov statistic on 2 samples: KS statistic = 0.067, pvalue = 0.07). (d-f) 2d trophallactic direction plots. Data included all interactions from the three experiments (N = 2141), and was binned according to the trophallactic-pair level of satiety as a fraction of the capacity of each ant (x-axis the an [file pcbi.1006925.s004.tif]

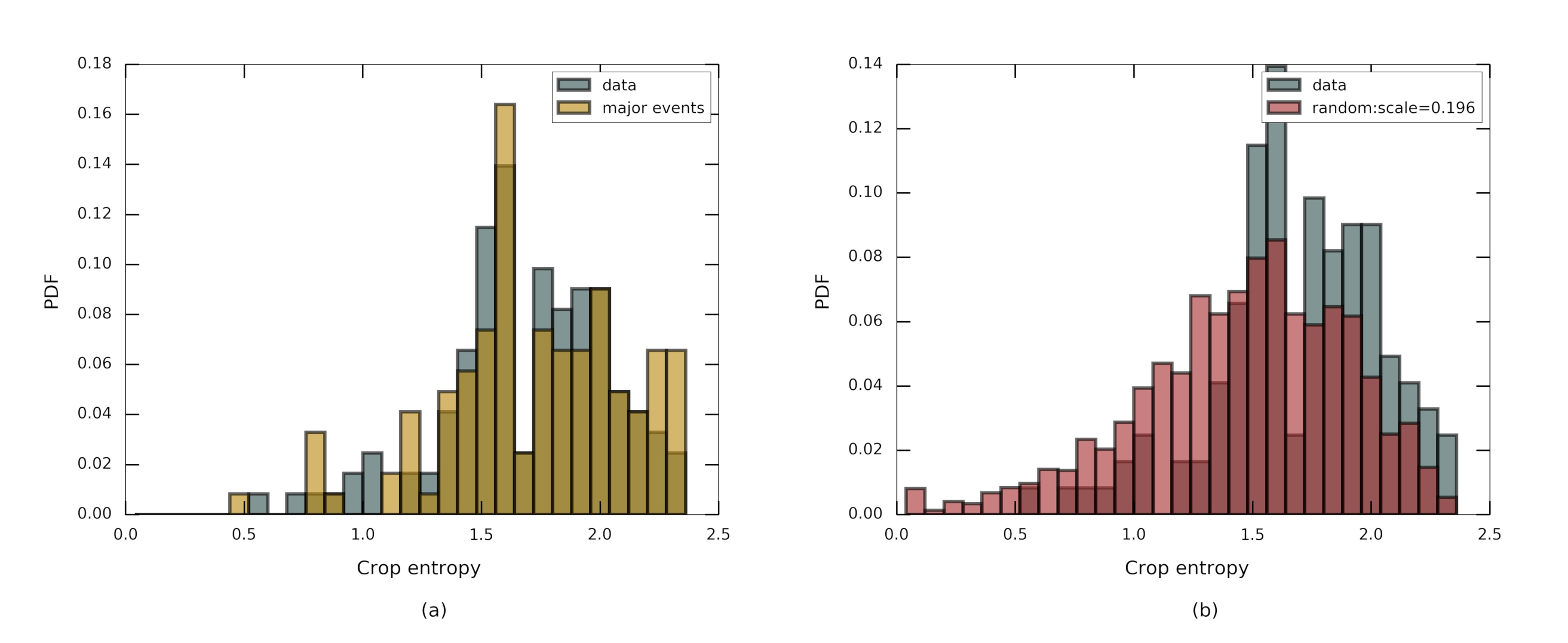

Supplement: S5 Fig — Relates to Fig 5. The number of largest events is chosen as the number of foragers in the experiment. a) PDF of crop entropy (grey) and the entropy generated by the largest events (yellow). Kolmogorov-Smirnov statistic on 2 samples: KS statistic = 0.15, pvalue = 0.13. b) PDF of crop entropy (grey) and the entropy generated by the sequence (red) m1, m2, ..Mn, where mj = δ(1 − δ)j−1, δ = ‘scale’ = 0.196 and n = number of foragers. Kolmogorov-Smirnov statistic on 2 samples: KS statistic = 0.01, pvalue = 0.57. (TIF) [file pcbi.1006925.s005.tif]
