# Supplementary material for: Colony entropy—Allocation of goods in ant colonies
Source: PLoS Comput Biol. 2019 Aug 5;15(8):e1006925. doi: 10.1371/journal.pcbi.1006925 (PMC6681937; doi:10.1371/journal.pcbi.1006925)
Supplement: S1 Text — Detailed methods and mathematical background. (PDF) [file pcbi.1006925.s007.pdf]

Colony entropy - Allocation of goods in ant colonies

Efrat Greenwald[1], Jean-Pierre Eckmann[2], Ofer Feinerman[1*]

1 Department of Physics of Complex systems, Weizmann Institute of Science, Rehovot, Israel.
2 Département de Physique Théorique and Section de Mathématiques, University of Geneva, Geneva, Switzerland.

* Corresponding author ofer.feinerman@weizmann.ac.il

Supplemental Information

SI Methods

Study Species: *Camponotus sanctus*

Camponotus sanctus are omnivorous ants that are presumed to naturally live in monogynous colonies of tens to hundreds of individuals (projecting from *Camponotus socius*, [1]), distributed from the near East to Iran and Afghanistan [2] . Workers of this species are relatively large (0.8-1.6 cm) and characterized by translucent gasters, rendering them suitable for both barcode labeling and crop imaging. Our experiments were conducted on lab colonies of 50-100 workers, reared from single queens that were collected during nuptial flights in Neve Shalom and Rehovot, Israel. Table A contains further details on each experimental colony.

Table A. Experimental Colonies.

**Colony**	**# Ants**	**Starvation**(weeks)	**# Major Workers***	**# Foragers**
A	100	3	1	5
B	62	5	1	3
C	53	4	2	4

* ‘Major Workers’ refers to the cast of workers in the colony which have distinctively larger body size.

Experimental setup

Fluorescent food imaging and 2D barcode identification (BugTag, Robiotec) were used to obtain a live visualization of the food flow through colonies of individually tagged ants. See [3] for a detailed description of the experimental setup. In short, an artificial nest was placed on a glass platform positioned between two cameras. A camera below the nest filmed through the platform, capturing the fluorescence emitted from the food inside the translucent ants. Meanwhile, a camera above the nest filmed through its infrared shelter, capturing the barcodes on the ants’ thoraxes, allowing identification of single ants inside the nest. Together, footages from both cameras enabled the association between each individual ant and her food load, throughout time and across trophallactic events. The two cameras were synchronously triggered at a fixed frame rate, (here 0.5 Hz., except for colony B which

was recorded at 1 Hz.). We chose a temporal resolution that is sufficient to capture events of 2 seconds since shorter interactions barely involve food exchange [3].

Image processing

Top camera images were used to extract ant identities, coordinates and orientations using the BugTag software (Robiotec). Bottom camera images were used to detect fluorescence with a pixel intensity threshold, using the openCV library in Python. Gasters of fed ants appeared as bright "blobs" and thus passed the image threshold (for details, see [3]).

In order to associate between the identity of an ant and her appropriate blob, the image from the upper camera was transformed to align with the fluorescent image. Then, for each identified tag, a small area extended from the back of the tag toward the ant's abdomen was crossed with the thresholded fluorescent image. If a blob intercepted this area, it was assigned to the tag's identity.

Thus, for each experiment a database was obtained, which included for every frame the coordinates, orientation, and measured fluorescence (in arbitrary units of pixel intensity) of each identified ant.

Experiment protocol

Following a food-deprivation period of 3-5 weeks, ant colonies (queen, workers and brood) were manually barcoded and introduced to the experimental nest for an acclimatization period of at least 4 hours. The nest consisted of an IR-sheltered chamber (~100 cm^2), neighboring an open area which served as a yard. After the acclimatization period, the two cameras synchronously started to record. After 30 minutes, the fluorescent food (sucrose [80 g/l], Rhodamine B [0.08 g/l]) was introduced to the nest yard *ad libitum*, and the recording proceeded for at least 4 more hours - a duration sufficient for the colony to reach its desired food volume intake [4].

Foragers

Each experiment consisted of a few individuals who performed consistent foraging cycles between the food source and the nest. Those ants were considered as "foragers". Some other individuals were occasionally observed at the food source but clearly did not display such foraging cycles. For our purposes they were not considered as foragers. These ants visited the food source no more than 4 times, while consistent foragers performed an average of 15.67 cycles and no less than 8. The data presented here is from the first return of a forager to the nest from the food source until the end of the experiment [4].

Interaction identification and crop load estimation

Even though the fluorescence emitted from an ant's crop is reasonably indicative of the food volume, it is a noisy measurement mainly due to her highly variable postures. Therefore, assuming that an ant's crop content remains constant during the intervals between trophallactic events, we evaluated the temporal food load by 90th percentile fluorescence measurement acquired in each such interval [3].

In order to precisely consider the relevant intervals for this estimation, the trophallactic interactions were manually identified from the video. Interactions were classified as trophallactic events whenever the mandibles of the participating ants came in contact and the mandibles of at least one of the ants were open. For forager ants, another situation in which their crop loads may change is when they directly feed from the food source. These feedings were also manually identified from the video, as times when a forager's open mandibles touched the food source.

The volume of trophallactic event

For each trophallactic event we recorded two measurements: one of each participating ant. While the transparency factors (calculated for each ant relative to the others [3]) reduce discrepancy between these two measurements they do not eliminate them completely. Thus, the amount of food transferred in an interaction n is calculated by a weighted average corresponding to a maximal likelihood of normal distribution:

$$m_n = \frac{\frac{\text{abs}(m_{i,n})}{\sigma^2_{i,n}} + \frac{\text{abs}(m_{j,n})}{\sigma^2_{j,n}}}{\frac{1}{\sigma^2_{i,n}} + \frac{1}{\sigma^2_{j,n}}} \quad (1)$$

Where: m_n - the estimate amount transferred in interaction n.

$m_{i,n}$ - is the amount transferred in interaction n according to ant i ($m_{i,n} = \frac{x_{i,n}}{\mu_i}$, $x_{i,n}$ - difference in pixels value according to ant i, μ_i - transparency factor associated with ant i see, [3]) $\sigma^2_{i,n}$ - measurement error (variance of the measurement points, standardized to the number of measurements in time intervals before and after the interaction) of $m_{i,n}$.

The interaction networks for all three colonies including interacting ant identities, timing, crop loads, and interaction volumes can be found in the accompanying file: S1 Data.

Simulations

Maximal mixing: the simulation follows an experimental interaction schedule (*i.e.*, pairs, time-order and direction) while 'equal sharing rule' is applied. In this case, at each interaction, each ant gives half of her own crop to her trophallactic mate. This is the best mixing scenario that can be achieved in a single interaction since both ants leave the interaction with identical crop loads. This interaction rule further translates to maximal mixing on the global level. Specifically, in each step of the simulation the maximal exchange rule is applied between the empirically determined interaction partners and their crop loads are accordingly updated (see above 'food tracking'). Finally, H_{mix} is calculated for the resulting food distributions .

Maximal transfer: the simulation follows an experimental interaction while 'maximal transfer rule' is applied. In this case, at each interaction, the experimental donor-ant gives the maximal amount that could have passed *i.e.*, min(donor's food load, recipient's capacity - recipient's food load) and H_{mix} is calculated as described above.

Foragers only: the simulation follows an experimental interaction schedule while only interactions that include foragers are taken into account. The transferred amount is taken from the experimental data and H_{mix} is calculated as described above.

Random rule: This simulation follows the time-order of pair-trophallaxis. In trophallaxis events between foragers and non-foragers the forager is assumed to be the donor, and in all other cases the donor is chosen randomly and with equal probably between the experimental pair. The transferred volume, $v = r \cdot v_p$, is determined by the multiplication of a random factor r ($r = \min(x, 1)$ where $p(x) = \frac{1}{\delta} \exp^{-\frac{x}{\delta}}$) and the potential volume v_p (v_p =min(donor's food load, recipient's capacity - recipient's food load)). H_{mix} is calculated as described above.

Shuffle simulation: We separately shuffle the donor time ordered-list and the recipients' list and create a new trophallaxis-network by combing these lists together (*i.e.*, the identity of the trophallaxis-mates, and hence the direction of the trophallaxis events are randomly changed). The transferred volume and H_{mix} are calculated as described for the random-rule above.

Trade-off simulation: . This simulation approximates food flows in the trade-off model as described in the main text. In this simulation all ants are assumed to have the same capacity. We begin each run (in total 30 runs per given $\tilde{\delta}$) by creating a random trophallaxis network that includes 60 ants in total, 4 foragers and 1000 interactions We use

a ratio of 3:1 between interaction that include foragers and ones that do not. These parameters of the simulations were chosen to approximate the empirical data in terms of number of ants, number of foragers, the average interaction per ant and the ratio between forager to non-forager and non-forager to non-forager interactions. At time zero, all ants are empty, except the foragers who are always completely full. The simulation follows the randomly generated network, and the transferred volume is taken to be a constant fraction, $\tilde{\delta}$, of the potential that could have passed (this is a deterministic approximation of the empirical random transfer rule described by the parameter δ). Here again, if a forager is involved in the trophallaxis she is taken as a donor, otherwise the donor is determined randomly. Each time step includes one trophallaxis event, in which the donor and transfer fraction $\tilde{\delta}$ out of the potential the could pass, $n_{a,f}(t)$ is reevaluated for trophallaxis mates. We use this data to calculate H_{mix} and $Pcol = \frac{\sum_{a \in \text{non-foragers}} n_a}{\text{total number of ants}*\text{capacity}}$.

Supplemental Text: Mathematical Framework

Table B. Entropy symbols

name	symbol
Colony entropy	$H_{\text{colony}}(P_{a,f}) = -\sum_{a\in\mathcal{A}} \sum_{f\in\mathcal{F}} P_{a,f} \log_2(P_{a,f})$
Overall dissemination entropy	$H_{\text{overall dissemination}} = -\sum_{a\in\mathcal{A}} P_a \log_2(P_a)$
Crop entropy	$h^a_{\text{mix}} \equiv H(F\|A=a) = \sum_{f\in\mathcal{F}} P_{f\|a} \log_2(P_{f\|a})$
Mixing entropy	$H_{\text{mix}} = \sum_{a\in\mathcal{A}} P_a h^a_{\text{mix}}$
Foragers' dissemination entropy	$H_{\text{foragers' dissemination}} = -\sum_{f\in\mathcal{F}} P_f \sum_{a\in\mathcal{A}} P_{a\|f} \log_2(P_{a\|f})$
Types entropy	$H_{\text{types}} = -\sum_{f\in\mathcal{F}}(\sum_{a\in\mathcal{A}} P_{f\|a}) \log_2(\sum_{a\in\mathcal{A}} P_{f\|a})$

Colony Entropy

In our experiments, only one food type was provided: a sucrose solution. Therefore, to study the mixing, we labeled each 'food droplet' according to the forager that brought it, and tracked it as it passed in trophallaxis (see Methods:'food tracking'). Denoting by:

$\mathcal{F} = \{1, 2, \ldots, N_{\text{foragers}} \equiv |\mathcal{F}|\}$ - the set of foragers in the colony.
$\mathcal{A} = \{N_{\text{foragers}}+1, N_{\text{foragers}}+2, \ldots, N_{\text{ants}}\}$- the set of non-forager workers in the colony
$n_{a,f}$ – number of food particles of type f held by ant a

The joint probability, $P_{a,f}$, that a particle of source f is in the crop of ant a :

$$P_{a,f} = \frac{n_{a,f}}{Z} \ . \tag{2}$$

where:

$$Z = \sum_{a\in\mathcal{A}} \sum_{f\in\mathcal{F}} n_{a,f} \ , \tag{3}$$

We also use

$$P_a = \sum_{f \in \mathcal{F}} P_{a,f} \ , \tag{4}$$

With this joint probability the total Entropy of the colony, H_{colony}, is defined as the Shannon Entropy (SI Fig S1):

$$H_{\text{colony}}(P_{a,f}) = -\sum_{a \in \mathcal{A}} \sum_{f \in \mathcal{F}} P_{a,f} \log_2(P_{a,f}) \tag{5}$$

$$\begin{aligned}
&H_{\text{colony}}(P_{a,f}) = -\sum_{a \in \mathcal{A}} \sum_{f \in \mathcal{F}} P_{a,f} \log_2(P_{a,f}) = \\
&-\sum_{a \in \mathcal{A}} \sum_{f \in \mathcal{F}} P_{f|a} P_a \log_2(P_{f|a} P_a) = \\
&-\sum_{a \in \mathcal{A}} \underbrace{\sum_{f \in \mathcal{F}} P_{f|a}}_{=1} P_a \log_2(P_a) - \sum_{a \in \mathcal{A}} \sum_{f \in \mathcal{F}} P_{f|a} P_a \log_2(P_{f|a}) = \\
&-\sum_{a \in \mathcal{A}} P(a) \log_2(P_a) - \sum_{a \in \mathcal{A}} P_a \underbrace{\sum_{f \in \mathcal{F}} P_{f|a} \log_2(P_{f|a})}_{h^a_{\text{mix}}} = \\
&H_{\text{overall dissemination}} + H_{\text{mix}}
\end{aligned} \tag{6}$$

and the following identities were used:

$$h^a_{\text{mix}} \equiv H(F|A=a) = \sum_{f \in \mathcal{F}} P_{f|a} \log_2(P_{f|a}) \tag{7a}$$

$$H_{\text{overall dissemination}} = -\sum_{a \in \mathcal{A}} P_a \log_2(P_a) \tag{7b}$$

$$H_{\text{mix}} = -\sum_{a \in \mathcal{A}} P_a h^a_{\text{mix}} \tag{7c}$$

$$\sum_{f \in \mathcal{F}} P_{f|a} = 1 \tag{7d}$$

$$P_{a,f} = P_{f|a} P_a \tag{7e}$$

$$P_{f|a} = \frac{n_{a,f}}{\sum_{f' \in \mathcal{F}} n_{a,f'}} = \frac{P_{f,a}}{P_a} \tag{7f}$$

$$P_a = \frac{\sum_{f' \in \mathcal{F}} n_{a,f'}}{\sum_{a' \in \mathcal{A}} \sum_{f' \in \mathcal{F}} n_{a',f'}} \tag{7g}$$

In a similar way, the entropy of the colony can also be divided into the two components, types and foragers' dissemination (SI Fig S1):

$$\begin{aligned}
&H_{\text{colony}}(P_{a,f}) = -\sum_{a \in \mathcal{A}} \sum_{f \in \mathcal{F}} P_{a|f} P_f \log_2(P_{a|f} P_f) = \\
&H_{\text{types}} + H_{\text{foragers' dissemination}}
\end{aligned} \tag{8}$$

where:

$$H_{\text{types}} = -\sum_{f\in\mathcal{F}} (\sum_{a\in\mathcal{A}} P_{f|a}) \log_2 (\sum_{a\in\mathcal{A}} P_{f|a}) \tag{9a}$$

$$H_{\text{foragers' dissemination}} = -\sum_{f\in\mathcal{F}} P_f \sum_{a\in\mathcal{A}} P_{a|f} \log_2 (P_{a|f}) \tag{9b}$$

Each of these four conditional entropies may be related to a different aspect of the collective level of the food dissemination process:

1. $H_{\text{overall dissemination}}$ - How uniform is the food divided across the workers of the colony (regardless the food type)?
2. H_{mix} - How well food is mixed across individuals?
3. H_{types} - The abundance of each food type
4. $H_{\text{foragers' dissemination}}$ - How uniform each food type is distributed across the ants?

Table B contains a summary of the different entropy definitions. For the dynamics of these different entropy terms please refer to figure Fig S1 ('Colony entropy').

Trade-off model

For the purpose of the model, we defined the amount of food held by a forager at time $t = 0$ to equal the total amount of food she collects at the food source during the entire course of the experiment. This definition sets the amount of food across all colony members, M, as a quantity that is conserved over time. Considering the entire colony we now define the probability $\tilde{P}_a = n_a(t)/M$ as the fraction of total amount of food held by any ant, forager or non-forager.

Using these definitions entails that at $t = 0$ all food is held by the foragers being, therefore, completely non-mixed while at later times, as food flows into the colony, it mixes within the crops of non-forager ants. This interplay between food accumulation and food mixing can be captured by considering the mixing entropy over all ants in the colony:

$$H_{\text{mix}}^{\text{overall}} = \sum_{a\in\mathcal{A}\cup\mathcal{F}} \tilde{P}_a h_{\text{mix}}^a.$$

Note that since foragers receive almost no food from other workers we can approximate $P(f'|a = f) \approx 1$ for $f' = f$ and zero otherwise. This means that $h_{\text{mix}}^f = 0$ for $f \in \mathcal{F}$ and leads to a second representation of $H_{\text{mix}}^{\text{overall}}$:

$$\begin{aligned} H_{\text{mix}}^{\text{overall}} &= \sum_{a\in\mathcal{F}\cup\mathcal{A}} \tilde{P}_a h_{\text{mix}}^a = \sum_{a\in\mathcal{F}} \tilde{P}_a h_{\text{mix}}^a + \sum_{a\in\mathcal{A}} \tilde{P}_a h_{\text{mix}}^a \\ &= (1 - P_{\text{colony}}) \cdot 0 + P_{\text{colony}} \cdot \sum_{a\in\mathcal{A}} \frac{\tilde{P}_a}{P_{\text{colony}}} h_{\text{mix}}^a \\ &= P_{\text{colony}} \cdot H_{\text{mix}} , \end{aligned}$$

where $P_{\text{colony}} = \sum_{a\in\mathcal{A}} \tilde{P}_a$ is the colony's satiation level which starts off at 0 and saturates at 1 as food flows into the system [3]. This representation, therefore, neatly separates the dissemination behavior into a component which quantifies the extent at which food is accumulated and a second component which quantifies the extent at which it is mixed.

Entropy by largest events

Having identified both the global mixing entropy and the local interaction rules, we aim to reveal how the former emerges from the later. Since the mixing entropy is the weighted average of the entropy of the food mixture within the individuals (h^a_{mix}), we examine the entropy induced by the largest-volume trophallactic events and a simplified food accumulation process inspired by the empirical 'δ rule'.

We find a high correlation between the crop entropy and the entropy calculated for the n-largest events (where n is taken as the number of foragers in the experiment a) the distributions of the crop entropy and entropy of the n largest interactions are similar (Kolmogorov-Smirnov statistic on 2 samples: KS statistic = 0.15, pvalue= 0.13, Fig S5(a)), meaning that the large interactions explain most of the crop entropy.

The δ rule suggests that, if not limited by the donor's food load, in a receiving interaction, an ant is provided with a random volume of food that follows an exponential distribution, with an average that is proportional to her free storage space (*i.e.*, the difference between her capacity and her current load [4]). This implies that, on average, an ant receives food in a series of decreasing volumes. Although this statement is correct only on average, for the estimation of the mixing level the order of the received quantities is not significant.

Assuming that an ant receives food in a sequence trophallactic interactions, each time of volume that equals to fraction δ of her free space, the amount of food received in the j-th interaction equals to:

$$m_j = \delta(1-\delta)^{j-1} \; ; \; j = 1, 2, ... \tag{10}$$

and further assuming that each interaction is of a different source type, the entropy of a sequence of n interactions is simply:

$$h^a_{\text{mix}} = -\sum_{j=1}^{n} -\frac{m_j}{M_n} \log_2(\frac{m_j}{M_n}) \tag{11}$$

where: $M_n = \sum_{j=1}^{n} m_j$

Fig S5(b) indeed shows that this very simplified accumulation model captures the main trend of the experimental outcome (Kolmogorov-Smirnov statistic on 2 samples: KS statistic = 0.01, pvalue= 0.57.)

References

1. Tschinkel WR. The nest architecture of the ant, Camponotus socius. Journal of Insect Science. 2005;5(1).

2. Ionescu-Hirsch A. An annotated list of Camponotus of Israel (Hymenoptera: Formicidae), with a key and descriptions of new species. Israel Journal of Entomology. 2009;39:57–98.

3. Greenwald E, Segre E, Feinerman O. Ant trophallactic networks: simultaneous measurement of interaction patterns and food dissemination. Scientific reports. 2015;5.

4. Greenwald EE, Baltiansky L, Feinerman O. Individual crop loads provide local control for collective food intake in ant colonies. eLife. 2018;7:e31730.

5. Hagberg A, Swart P, S Chult D. Exploring network structure, dynamics, and function using NetworkX. Los Alamos National Lab.(LANL), Los Alamos, NM (United States); 2008.
